# RNA polymerase II phosphorylation dynamics: from molecular mechanisms to human disease

**DOI:** 10.1080/15476286.2026.2695549

**Published:** 2026-06-29

**Authors:** Araceli González-Jiménez, Ithaisa Medina, Manuel J. Alfonso, Carlos R. Vázquez de Aldana, Olga Calvo

**Affiliations:** Instituto de Biología Funcional y Genómica (IBFG), CSIC-USAL, Salamanca, Spain

**Keywords:** RNAPII, phosphorylation, transcriptional regulation, neurodegeneration, cancer, therapeutic targeting

## Abstract

Accurate RNA polymerase II (RNAPII)–dependent gene expression requires dynamic phosphorylation of the carboxy-terminal domain (CTD) of its largest subunit, Rpb1, whose heptapeptide repeats form a regulatory platform known as the CTD code. Transcription-associated cyclin-dependent kinases (tCDKs) and CTD phosphatases coordinate the phosphorylation – dephosphorylation cycle of RNAPII throughout transcription, coupling RNA synthesis to co-transcriptional processing and chromatin regulation. By controlling stage-specific modification of the CTD, these enzymes integrate RNAPII activity into broader regulatory networks. Disruption of the delicate kinase – phosphatase balance impairs transcriptional fidelity, RNA maturation, and genome stability, either directly through altered CTD phosphorylation or indirectly through associated pathways. Such alterations are increasingly associated with developmental disorders, neurodegeneration, and cancer. Here, we synthesize current knowledge of RNAPII phosphorylation dynamics, highlighting key mechanistic principles, links to human disease, and emerging therapeutic strategies targeting this central phosphorylation-dependent regulatory system.

## Introduction

The biogenesis of RNA polymerase II (RNAPII)-transcribed RNA requires precise coordination between RNA synthesis, co-transcriptional RNA processing, and chromatin modifications. The coordination is mediated by reversible phosphorylation of the carboxy-terminal domain (CTD) of the RNAPII largest subunit, Rpb1—an intrinsically disordered region composed of tandem heptapeptide repeats. Phosphorylation of specific CTD residues generates stage-specific patterns – termed the ‘*CTD code’* that direct factor recruitment, coupling transcription to RNA maturation events [1,2]. More broadly, protein phosphorylation represents one of the most widespread post-translational modifications across evolution and all domains of life, supporting the emergence of regulatory networks required for cellular complexity [3]. The phosphorylation of Rpb1-CTD exemplifies how phosphorylation-based signalling has been adapted to control dynamic, multi-step processes such as eukaryotic gene expression.

The CTD phosphorylation landscape is established by transcription-associated cyclin-dependent kinases (tCDKs) and counterbalanced by CTD-specific phosphatases, creating a dynamic equilibrium that governs RNAPII progression through chromatin during the transcription cycle [1,2,4,5]. Moreover, it ensures the correct timing of RNA processing, chromatin remodelling, and transcription termination [2].

Disruption of the kinase – phosphatase balance – through loss-of-function mutations, altered expression, or aberrant signalling pathways affecting CTD-modifying enzymes – produces altered CTD phosphorylation patterns that compromise transcriptional fidelity, RNA processing and passage of the RNAPII through the nucleosomes [6–10]. Beyond direct effects on the CTD, many of these enzymes also phosphorylate or dephosphorylate additional substrates – including transcription factors, splicing regulators and signalling proteins – thereby extending their pathological impact to broader transcriptional and cellular networks. In fact, disruption of the phosphorylation – dephosphorylation balance, even at a subtle level, can perturb gene-expression programmes, compromise genome stability and alter cellular identity, leading to severe pathological outcomes, such as developmental disorders, neurodegeneration, and cancer [2,11–13]. This highlights the essential requirement for finely tuned kinases – phosphatases coordination in maintaining transcriptional homeostasis.

This review delineates the molecular mechanisms governing CTD phosphorylation and the pathophysiological repercussions of its dysregulation. Special emphasis is placed on CTD-regulatory proteins and the broader phosphorylation networks in which they act as disease drivers and therapeutic targets, illustrating the translational potential of modulating this post-translational scaffold.

## Molecular architecture of RNA polymerase II and its phosphorylation landscape

In eukaryotic cells, RNAPII is a twelve-subunit (Rpb1-Rpb12) enzyme responsible for the synthesis of all messenger RNAs (mRNAs) and several classes of non-coding RNAs (ncRNAs). Its core catalytic activity resides in the two largest subunits, Rpb1 and Rpb2, which form a central cleft that accommodates the DNA – RNA hybrid during transcription [14–16]. The remaining subunits are organized into peripheral modules that stabilize the complex and mediate interactions with regulatory factors [14–18]. The core module includes an assembly platform composed of Rpb3, Rpb10, Rpb11, and Rpb12, which anchors the large subunits and is essential for enzymatic activity [14,16,18]. The jaw-lobe module (Rpb1, Rpb5, Rpb9) and shelf module (Rpb1, Rpb5) jointly direct DNA towards the active centre and stabilize the transcription complex [18]. The stalk module, formed by Rpb4 and Rpb7 subunits positioned near the RNA exit channel and the Rpb1-CTD, plays a key role in coupling transcription with mRNA processing and export [14,19].

**Figure 1.**
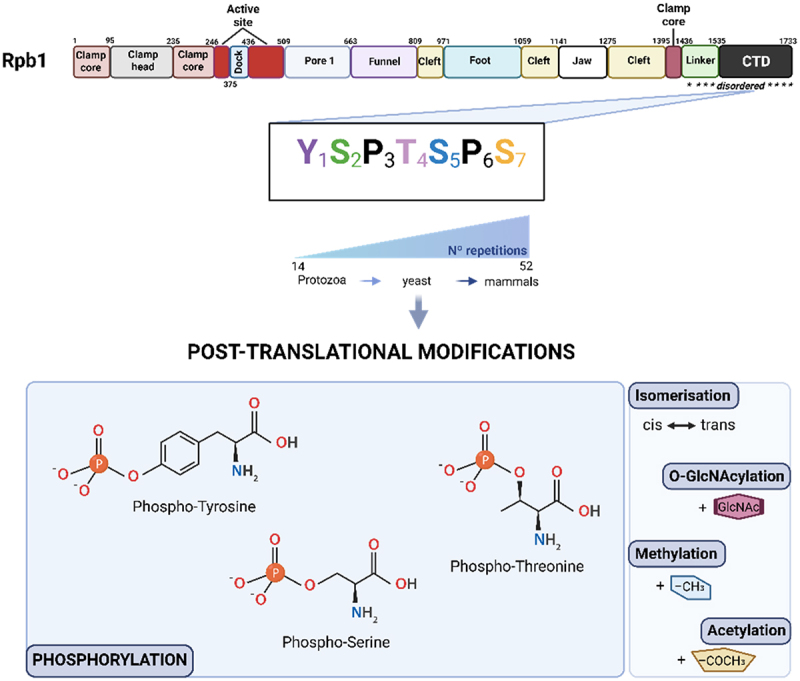
Structure of RNA polymerase II subunit Rpb1 and post-translational modifications of its CTD. Schematic representation of *S.* *cerevisiae* Rpb1 functional domains, highlighting the CTD. The CTD consists of tandem repeats of the conserved heptapeptide consensus sequence Y_1_S_2_P_3_T_4_S_5_P_6_S_7_, whose number increases from protozoa to yeast and mammals. Phosphorylation constitutes the predominant CTD modification, with additional regulatory roles reported for proline isomerisation, O-GlcNAcylation, methylation, and acetylation.

A unique feature of RNAPII is the CTD of Rpb1 (Figure 1), an unstructured domain, which extends from the catalytic core towards the RNA exit channel serving as a platform for the binding of several factors and therefore acts as a dynamic regulatory interface coordinating RNA metabolic processes (transcription and RNA maturation) and chromatin-based processes [20–22]. Binding of factors to the CTD is primarily regulated by phosphorylation/dephosphorylation of an heptapeptide consensus sequence Tyr1-Ser2-Pro3-Thr4-Ser5-Pro6-Ser7 (Y_1_S_2_P_3_T_4_S_5_P_6_S_7_ [23]), evolutionarily conserved, and tandemly repeated in a number that varies with genome complexity [5]. The number of CTD heptad repeats ranges from approximately 14 repeats in protozoa, and 26–29 in *Saccharomyces cerevisiae* to 52 in mammals, reflecting the expanded regulatory demands of higher eukaryotes [5]. Whilst yeast CTD repeats are largely uniform, higher eukaryotes display greater sequence divergence, with mammals possessing a highly conserved N-terminal region followed by more variable C-terminal repeats [24]. Although dispensable for basal catalytic activity *in vitro*, the CTD is essential for cell viability and serves as a crucial platform for orchestrating mRNA biogenesis by coupling transcription with co-transcriptional processing events, including 5’capping, splicing, and 3’ polyadenylation [2].

This regulatory function is mediated principally by extensive phosphorylation of residues within the CTD heptad repeats, which generates a dynamic CTD code that recruits RNA-processing, histone-modifying, and chromatin-remodelling factors in a transcription stage-dependent manner [1,4,5]. The predominant modifications involve coordinated phosphorylation of Ser5, Ser7, and Ser2 residues (Figure 1). CTD-Ser5P peaks at initiation near the transcription start site, CTD-Ser7P is deposited concurrently with Ser5 and persists across many transcribed regions, and CTD-Ser2P accumulates progressively during elongation and reaches maximal levels near the polyadenylation signal to coordinate 3′-end formation [2,5]. Additional phosphorylation events further expand CTD regulatory capacity. CTD-Tyr1P prevents premature termination and supports productive elongation and enhancer activity [25–27], whereas CTD-Thr4P functions in snoRNA transcription termination, elongation of protein-coding genes, and histone mRNA 3′-end processing [28,29]. In higher eukaryotes, CTD-Ser7P recruits the Integrator complex to snRNA genes [30]. Although phosphorylation constitutes the canonical CTD code, additional PTMs – including O-GlcNAcylation, methylation and acetylation – further modulate CTD regulatory capacity (Figure 1 [31]).

Beyond the CTD, emerging evidence shows that regulatory phosphorylation extends to non-CTD regions of Rpb1, and to other RNAPII subunits, indicating additional layers of transcriptional control. In yeast, Rpb1 undergoes phosphorylation at T1471/S1493 and Y1473/S1493 within the linker region – a domain connecting the CTD to the polymerase core – which is essential for recruiting the elongation factor Spt6 and for maintaining repressive chromatin states [32]. The linker phosphorylation, catalyzed by Bur1 in yeast and by CDK9 in human cells, promotes RNAPII release from promoter-proximal pausing [33,34]. Additionally, proteomic studies have revealed numerous phosphorylation sites across all RNAPII subunits in humans, and although yeast shows a more limited distribution – with Rpb7 and Rpb11 being the only subunits lacking detectable phospho-residues – the functional significance of most phosphorylation events, aside from those in the CTD, remains largely unresolved [35]. These findings indicate that RNAPII phosphorylation regulation extends beyond the canonical CTD phospho-code to encompass a broader network of post-translational modifications distributed throughout the RNAPII complex.

## Kinase–phosphatase dynamics governing RNA polymerase II phosphorylation

**Figure 2.**
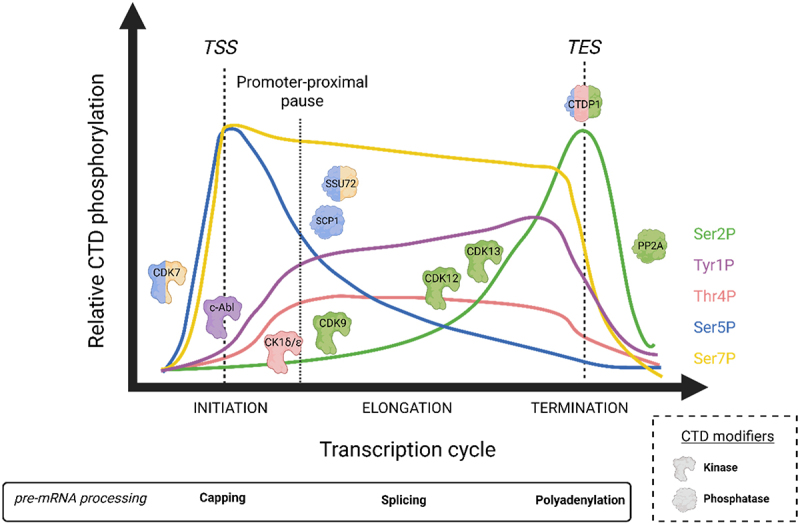
Dynamic regulation of human Rpb1-CTD phosphorylation during the transcription cycle. CTD kinases (CDK7, c-abl, CK1δ/ε, CDK9, CDK12, CDK13) and CTD phosphatases (SSU72, SCP1, CTDP1, PP2A) regulate specific CTD residues phosphorylation/dephosphorylation throughout the transcription cycle. The sequential addition and removal of phosphate marks by kinases and phosphatases, respectively, creates a ‘CTD code’ that recruits specific factors for co-transcriptional mRNA processing events, including capping, splicing, and polyadenylation: phosphorylation levels of Ser2 (green), Tyr1 (purple), Thr4 (red), Ser5 (blue), and Ser7 (yellow) vary dynamically across the transcription cycle. Ser5 and Ser7 phosphorylation peak at initiation near the transcription start site (TSS), facilitating RNA capping. Ser2 phosphorylation accumulates across the gene body during elongation, where it contributes to co-transcriptional splicing, and peaks near the transcription termination site (TES), coordinating 3’-end processing.

The phosphorylation landscape of RNAPII is established and maintained through the coordinated activity of evolutionarily conserved transcription-associated kinases – primarily CDKs (CDK7, CDK9, CDK12 and CDK13) – and CTD-directed phosphatases, including FCP1 and SSU72 (Figure 2). Together, these enzymes orchestrate the successive phosphorylation and dephosphorylation events that generate the stage-specific CTD patterns coupling transcription – from initiation through elongation and termination – to RNA processing, histone modification, chromatin remodelling and mRNA export (Figure 2 [1,4,20,36]). The mechanistic framework for this regulatory cycle, originally established in *S. cerevisiae* [29,37–39], has expanded substantially in metazoans, where additional kinases and signalling pathways confer increased versatility to support complex, cell-type-specific transcriptional programmes [2,5,40]. Recent evidence suggests that the repertoire of CTD kinases is substantially broader than previously appreciated. A large-scale kinome-wide screen identified 117 human kinases capable of phosphorylating one or more CTD residues *in vitro*, and validated selected receptor tyrosine kinases, including EGFR, as CTD kinases *in vivo* [40]. These findings considerably expand the traditional view of CTD regulation and establish RNAPII as a direct target of signalling pathways.

During transcription initiation, the earliest CTD phosphorylation events are catalysed by CDK7, the catalytic subunit of the general transcription factor TFIIH. CDK7 phosphorylates Ser5 and Ser7 residues near the transcription start site, promoting promoter clearance and recruitment of the mRNA-capping enzyme (Figure 2), which specifically recognizes the CTD-Ser5P mark to initiate co-transcriptional 5′-cap formation [41]. The orthologous TFIIH kinase Kin28 in yeast performs a similar promoter-proximal function, establishing the early CTD-Ser5P pattern required for promoter escape and capping-enzyme recruitment [42,43]. CTD-Ser5 phosphorylation also recruits the Set1/COMPASS methyltransferase complex to deposit the H3K4me3 histone mark characteristic of active promoters [44,45]. Concurrently, Ser7 phosphorylation, likewise mediated by CDK7, has a well-established role in RNAPII – dependent snRNA 3′-end processing [30,46], but also contributes to promoter-proximal pausing and transcription termination at mRNA genes [47]. CDK8, operating within the Mediator kinase module, further modulates RNAPII engagement with the pre-initiation complex through regulation of Mediator activity and phosphorylation of transcription factors (Figure 2), thereby fine-tuning transcriptional output in response to developmental and environmental cues [48].

As RNAPII transitions from initiation to productive elongation, phosphatases remodel the CTD landscape to shift the balance of modifications. In metazoans, the small CTD phosphatase 1 (SCP1/CTDSP1) has been reported to dephosphorylate CTD-Ser5P *in vitro*, although direct evidence supporting a major physiological role in CTD regulation during the transcription cycle remains limited (Figure 2 [49,50]). SSU72, an evolutionarily conserved aspartate-based CTD phosphatase (Ssu72 in yeast), whose inactivation increases CTD-Ser5P and CTD-Ser7P levels [51,52], plays a central role in Ser5 dephosphorylation during the initiation to elongation transition (Figure 2 [38,53–55]). In both yeast and mammals, SSU72 also contributes to transcription termination and RNA 3′-end processing [38,51]. Specifically, in mammals, structural and functional studies have shown that SSU72 cooperates with Symplekin and is required for efficient 3′-end formation, and that SSU72 inactivation impairs mRNA and snRNA processing [51,52]. However, unlike in yeast, mammalian SSU72 does not appear to be a stable component of the cleavage and polyadenylation machinery, suggesting a more indirect role mediated through CTD phosphorylation dynamics [39,56].

Once the polymerase escapes promoter-proximal pausing, productive elongation is driven by CDK9, the catalytic subunit of positive transcription elongation factor b (P-TEFb). CDK9 phosphorylates CTD-Ser2 and, to a lesser extent, CTD-Ser5 and CTD-Ser7 both *in vitro* and *in vivo* [57,58], as well as the elongation factors DSIF (SPT4/SPT5) and NELF, thereby releasing paused polymerases into productive elongation [59,60]. CDK12 and its paralogue CDK13 maintain CTD-Ser2P during late elongation, coordinating co-transcriptional splicing with RNA 3′-end formation (Figure 2), and sustaining transcription of long genes involved in DNA repair and genome stability [61–63]. In budding yeast, analogous elongation-associated functions are performed by Bur1 and Ctk1, with Ctk1 acting as the major CTD-Ser2 kinase across gene bodies and towards gene 3′ ends [37,64–66]. Ser2 phosphorylation gradually accumulates towards the 3′ end of genes, promoting the recruitment of splicing and 3′-end processing factors and coupling transcription elongation to RNA maturation and termination (Figure 2 [64,65,67,68]).

Beyond serine phosphorylation, additional residues contribute to transcriptional regulation. CTD-Tyr1 phosphorylation accumulates across gene bodies, where it prevents premature termination by inhibiting recruitment of termination factors and promotes productive elongation through engagement of the elongation factor SPT6 [26]. In mammals, CTD-Tyr1P mediated by the c-Abl kinase modulates P-TEFb substrate specificity, enhancing CTD-Ser2P levels and thereby facilitating the transition from initiation to productive elongation [69,70]. CTD-Thr4 phosphorylation, mediated by casein kinase 1 family members (CK1δ/ε in mammals, Hrr25 in yeast), rises towards the gene 3′-end, facilitating transcription termination (Figure 2), particularly of non-coding RNA genes [29,71]. Recent genome-wide analyses further support the view that CTD-Thr4 phosphorylation represents a conserved feature of transcription termination [72].

As RNAPII approaches the 3’-end of genes, dephosphorylation resets the CTD for recycling and subsequent rounds of transcription (Figure 2). The canonical phosphatase CTDP1 (FCP1, TFIIF-associating CTD phosphatase 1), the major CTD phosphatase in higher eukaryotes, primarily targets CTD-Ser2P during elongation and termination, thereby promoting RNAPII release from chromatin and restoration of the hypo-phosphorylated form competent for re-initiation [73]. Although FCP1 can dephosphorylate both Ser2P and Ser5P *in vitro*, its physiological preference for Ser2P appears to be conserved from yeast to mammals [71]. FCP1 activity is itself regulated by phosphorylation, which modulates both its CTD phosphatase and transcription elongation activities [74].

Additional phosphatases contribute to CTD regulation in metazoans. RPAP2, initially characterized as a CTD-Ser5/Ser7 phosphatase [46], has been more recently proposed to primarily facilitate RNAPII loading onto promoters rather than act as a major CTD phosphatase during transcription [75]. Broader serine/threonine phosphatases, such as PP2A, associate with the metazoan-specific Integrator complex (not found in yeast) to form the INTAC complex, which dephosphorylates paused RNAPII and regulates promoter-proximal pause-release and termination [76,77]. Additionally, PP1 phosphatase, acting through the PNUTS phosphatase complex, also contributes to RNAPII CTD dephosphorylation and transcription termination in mammalian cells [78]. Recent evidence further demonstrates that coordinated CDK9–PP2A interplay within INTAC fine-tunes pause-release and termination dynamics, highlighting the tight coupling of kinase and phosphatase activities [79].

Together, CTD kinases and phosphatases activities establish a self-sustaining phosphorylation – dephosphorylation cycle that acts as the molecular engine of transcription.

Recent biophysical studies reveal that the CTD itself contributes to this regulatory logic. The aromaticity of Tyr1 and the conformational plasticity conferred by the *cis/trans* isomerization of proline residues influence CTD folding, phase-separation behaviour, and accessibility to CTD-modifying enzymes [80]. Prolyl isomerization, catalyzed by the peptidyl-prolyl isomerase Pin1, is now recognized as a critical determinant of CTD dephosphorylation dynamics: via *cis/trans* isomerization of phospho-Ser/Thr – Pro motifs, Pin1 controls substrate recognition by CTD phosphatases and thereby modulates transcriptional output, with profound implications for cancer biology [81,82]. Structural regulation is not limited to the CTD: the Rpb7 subunit recruits CTDP1 to the core enzyme to promote CTD-Ser2P dephosphorylation and RNAPII recycling [83]. Consistent with this model, work from our group has shown that Sub1 (PC4 in humans) interacts with the Rpb4/7 stalk to modulate Fcp1 access to the Rpb1 CTD, coupling elongation dynamics with phosphatase activity [84,85]. All these findings reinforce the concept that CTD regulation arises from the interplay of enzymatic, structural, and biophysical mechanisms; and underscore that CTD phosphorylation control is supported not only by networks of kinases and phosphatases, but also by the conformational and organizational properties of RNAPII, reflecting a multi-layered regulatory system.

## Dysregulation of RNA polymerase II phosphorylation in human disease

Disruption of RNA polymerase II phosphorylation dynamics has emerged as a recurrent feature of diverse human pathologies. Alterations in transcription-associated kinases and phosphatases can perturb gene-expression programmes through both CTD-dependent and CTD-independent mechanisms, affecting transcriptional elongation, RNA processing, genome stability, and cellular identity. While many disease-associated phenotypes reflect broader phosphorylation network dysregulation, a smaller subset of disorders arises from primary defects in CTD phosphorylation homoeostasis. Distinguishing between these mechanistic layers is essential for understanding the basis of transcription-associated disease and for designing therapeutic strategies targeting the RNAPII phosphorylation network.

## Primary disorders of Rpb1-CTD phosphorylation homeostasis

Primary disorders of CTD phosphorylation homeostasis are characterized by direct impairment of the enzymatic machinery responsible for establishing or resetting CTD phosphorylation patterns. In these conditions, defective control of CTD modification disrupts the transcription cycle at a fundamental level, compromising RNAPII recycling, elongation dynamics, and co-transcriptional RNA processing. Although rare, such disorders provide compelling evidence that precise CTD phosphorylation balance is indispensable for human development and tissue homeostasis.

**Figure 3.**
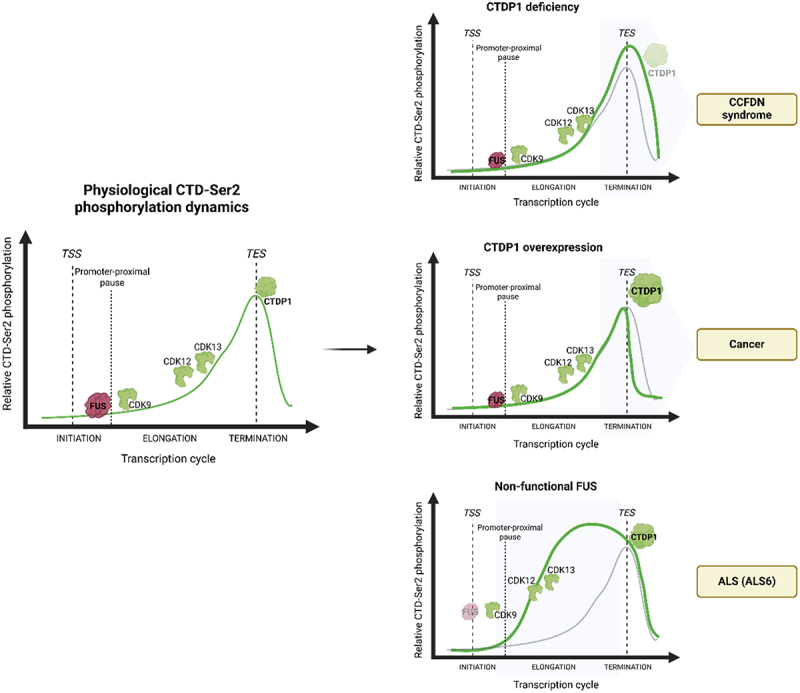
Primary disorders of CTD phosphorylation homeostasis. Schematic representation of relative CTD-Ser2 phosphorylation levels across the transcription cycle under physiological and pathological conditions. Left, physiological CTD-Ser2 phosphorylation dynamics during transcription initiation, elongation, and termination, coordinated by CTD kinases, phosphatases, and regulatory CTD-interacting proteins. Right, primary perturbations of CTD phosphorylation homeostasis. The green curve shows the pattern of CTD-Ser2P disrupted under each pathological condition, while the light gray curve corresponds to the canonical levels of CTD-Ser2P under physiological conditions. Top, CTDP1 deficiency, as observed in congenital cataracts facial dysmorphism neuropathy (CCFDN) syndrome, leads to impaired CTD dephosphorylation at transcription termination, resulting in persistent Ser2 hyperphosphorylation and defective RNAPII recycling. Middle, CTDP1 overexpression, reported in cancer, disrupts the dynamic balance of CTD phosphorylation, altering transcriptional homeostasis. Bottom, loss of functional FUS, associated with amyotrophic lateral sclerosis (ALS6), impairs CTD-dependent regulation of transcription elongation by relieving inhibition of CDK9/CDK12, leading to aberrant Ser2 hyperphosphorylation, promoter-proximal RNAPII accumulation, and premature transcription termination.

Among the CTD-specific phosphatases, FCP1 (encoded by *CTDP1*) represents the most extensively characterized and clinically relevant enzyme. Complete Ctdp1 deficiency in mice causes early embryonic lethality, whilst reduced activity disrupts RNAPII recycling and impairs transcription of genes requiring efficient CTD turnover [86]. Genetic ablation of CTDP1 causes congenital cataracts facial dysmorphism neuropathy (CCFDN) syndrome (Figure 3), a rare autosomal recessive disorder predominantly found in the Roma population [13]. All affected individuals are homozygous for a founder mutation (c.863 + 389C > T) in an intronic antisense Alu element within intron 6, which disrupts splicing and inserts a 95-nucleotide Alu sequence into the processed mRNA, causing premature termination [13,87]. This mutation reduces wild-type transcript levels to 15–35% of controls, resulting in partial protein deficiency [13]. To date, no studies have directly examined the effect of the disease-linked founder mutation on CTD phosphorylation dynamics in patient-derived cells or in cellular/organismal models carrying the same mutation. The link between CCFDN and CTD phosphorylation therefore remains largely inferential, based on the established enzymatic function of FCP1 as a CTD phosphatase and on mouse models of Ctdp1 deficiency [86]. Clinical features include bilateral congenital cataracts with microcornea, progressive hypo-/demyelinating peripheral neuropathy, cerebellar involvement (ataxia, nystagmus, intention tremor, dysmetria), mild-to-moderate intellectual disability, short stature, skeletal deformities, and hypogonadotropic hypogonadism [87,88]. Neuroimaging demonstrates cerebral and spinal cord atrophy [88,89]. CCFDN is considered a ‘transcription syndrome’- one of the very few known disorders directly involving defects in RNAPII-mediated gene expression [87] . On the other hand, as shown in Figure 3, CTDP1 overexpression in epithelial ovarian cancer correlates with poor prognosis, increased tumour immune infiltration, and adverse clinical outcomes [9]. Thus, both CTDP1 deficiency (CCFDN) and overexpression (ovarian cancer) disrupt transcriptional homeostasis, highlighting its relevance in developmental pathology and cancer biology.

Another disorder directly linked to perturbation of CTD phosphorylation dynamics involves mutations in CTD-interacting regulatory proteins rather than the modifying enzymes themselves [2]. These proteins recognize specific CTD phosphorylation patterns, modulate CTD modification states, or couple CTD dynamics to downstream processes such as pre-mRNA processing and transcription termination, thereby influencing transcriptional output in a phosphorylation-dependent manner.

The RNA-binding protein FUS (fused in sarcoma) exemplifies how CTD-interacting proteins link transcriptional regulation to neurodegenerative disease. Autosomal dominant mutations in FUS cause approximately 0.3–0.9% of amyotrophic lateral sclerosis (ALS) cases, classified as ALS type 6 (ALS6), and typically present with aggressive and early-onset disease (Figure 3 [90,]). Through direct binding to the Rpb1-CTD, FUS restrains aberrant Ser2 hyperphosphorylation by inhibiting CDK9 and CDK12 kinase activity [91]. Loss of functional FUS disrupts this regulation, resulting in excessive CTD-Ser2P that paradoxically impairs productive elongation: RNAPII accumulates at the promoter-proximal regions whilst failing to efficiently progress through gene bodies, leading to premature cleavage and polyadenylation at cryptic upstream sites rather than canonical 3’-end processing signals [91]. The pathogenic mechanisms involve loss of nuclear FUS function in regulating CTD phosphorylation and pre-mRNA processing, toxic cytoplasmic aggregation disrupting mRNA homeostasis and stress granule dynamics, as well as impaired DNA damage response and mitochondrial function [92]. Importantly, the dysregulation of CTD-Ser2 phosphorylation by mutant FUS has been directly demonstrated in ALS patient-derived fibroblasts, where loss of FUS function in orchestrating Ser2 phosphorylation is detectable even when FUS protein remains largely nuclear [93]. This provides direct evidence from patient-derived cells that mutant FUS is associated with altered CTD-Ser2 phosphorylation dynamics, strengthening the link between defective CTD regulation and FUS-associated ALS [93].

Another RNA-binding protein central to ALS, TDP-43 (encoded by TARDBP), has also been linked to transcriptional dysregulation and RNAPII-dependent gene expression. TDP-43 cytoplasmic aggregation is the pathological hallmark of most sporadic ALS cases, and its extensive functional overlap with FUS in RNA metabolism, transcriptional regulation, and neurodegeneration further supports the concept that disruption of CTD-associated transcriptional networks contributes to ALS pathogenesis [94].

## Transcription-associated CDKs as drivers of phosphorylation network dysregulation

Transcription-associated CDKs, such as CDK7, CDK8, CDK9, CDK12, and CDK13, have emerged as pivotal regulators at the intersection of signalling, transcription and genomic stability. Acting through phosphorylation, they govern transcription, from initiation to termination, and modulate the activity and recruitment of transcription factors [95]. Although these kinases phosphorylate the CTD as part of their canonical function, their pathological impact predominantly reflects dysregulation of RNAPII-centred transcriptional programmes rather than isolated CTD phospho-code imbalance.

**Figure 4.**
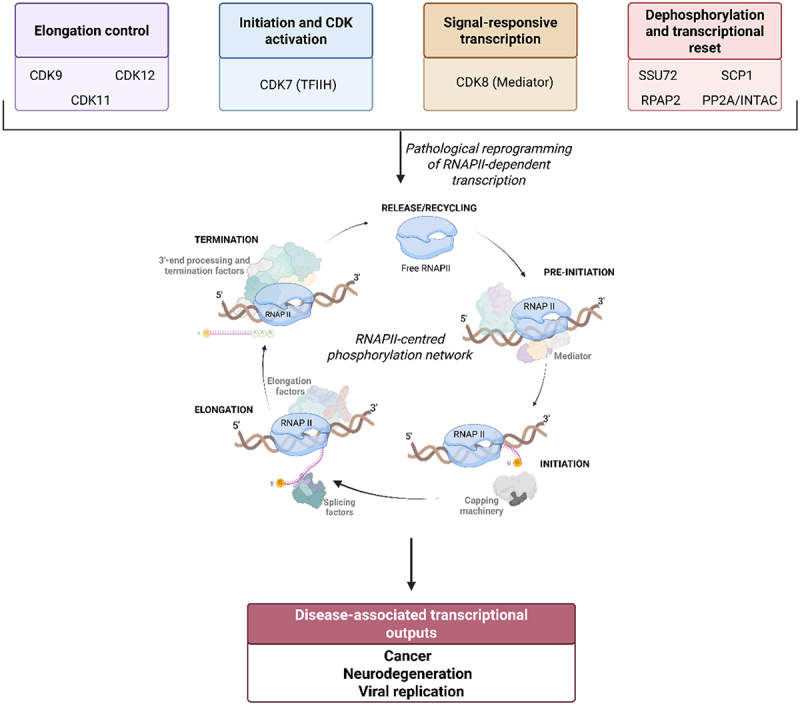
RNAPII-centred phosphorylation network dysregulation and disease-associated transcriptional reprogramming. Schematic overview of how transcription-associated kinases and phosphatases converge on RNAPII to reprogramme transcriptional outputs in disease. Top, functional modules of transcription-associated CDKs and CTD phosphatases, grouped according to their primary roles in elongation control (CDK9, CDK11, CDK12), transcription initiation and CDK activation (CDK7/TFIIH), signal-responsive transcription (CDK8/Mediator), and CTD dephosphorylation and transcriptional reset (SSU72, SCP1, RPAP2, PP2A/INTAC). Middle, the RNAPII transcription cycle, illustrating RNAPII as a central integration hub for phosphorylation-dependent regulatory inputs that coordinate initiation, elongation, termination, and recycling. Bottom, disruption of these RNAPII-centred phosphorylation networks leads to disease-associated transcriptional reprogramming, contributing to pathological outcomes including cancer, neurodegeneration, and viral replication.

Among these kinases, CDK9 exemplifies the intersection between transcriptional regulation and oncogenic signalling (Figure 4). Through phosphorylation of paused RNAPII elongation complexes, CDK9 enables efficient transcription by recruiting elongation factors such as Spt6 and PAF. This activity directly sustains oncogenic transcriptional programmes, most notably those driven by the transcription factor MYC, which is dysregulated in approximately 70% of cancers [96]. Oncogenic mutations often place MYC under the control of super-enhancers, creating a feedback loop where CDK9-driven transcriptional hyperactivation of MYC promotes further recruitment of CDK9 and stabilization of the MYC protein itself through phosphorylation at Ser62 [96,97]. In the liver, shifts in CDK9 isoform ratios (CDK942/CDK955) promote MYC-driven hepatocarcinogenesis [98].

In addition to MYC-related oncogenesis, CDK9 contributes to tumorigenesis by modulating p53, allowing genetically damaged cells to proliferate. CDK9 deficiency reduces p53 activity, elevates NPAT-mediated histone transcription, and disrupts DNA repair, promoting malignant progression [99]. In haematologic cancers, elevated CDK9/Cyclin T1 and MLL – EAP fusion complexes aberrantly sustain P-TEFb activity and block haematopoietic differentiation [12]. Beyond cancer, CDK9 also plays a pivotal role in the interaction between viral proteins and the transcription machinery during HIV-1 infection. As part of the P-TEFb complex with cyclin T, CDK9 is essential for HIV-1 transcriptional elongation driven by the viral Tat protein, which recruits cyclin T – CDK9 to TAR RNA to potentiate RNAPII activity [100]. Limiting or inhibiting P-TEFb activity suppresses Tat-activated gene expression and viral replication without broadly impairing host transcription, highlighting CDK9 as a critical host factor for HIV-1 propagation [101].

CDK8, as part of the Mediator kinase module, modulates RNAPII activity in a context-dependent manner, fine-tuning transcriptional responses [102,103]. In basal states, CDK8 primarily regulates transcription through phosphorylation of transcription factors and modulation of Mediator activity. By influencing Mediator conformation and RNAPII engagement, CDK8 can act as a transcriptional brake that limits reinitiation under specific regulatory contexts [48]. Conversely, under stress conditions, CDK8 cooperates with HIF1A to activate hypoxia-responsive transcriptional programmes [48]. The Mediator kinase module also integrates inflammatory and senescence pathways, as CDK8 drives transcription of SASP genes in chondrocytes in cooperation with NF-κB, promoting inflammatory microenvironments that exacerbate osteoarthritis [104–106]. Moreover, CDK8 amplification has been identified in colorectal cancer (Figure 4), where it potentiates β-catenin/TCF-dependent transcription of key oncogenes such as MYC and LEF1, correlating with poor prognosis [105,107].

As a component of the TFIIH complex, CDK7 phosphorylates Rpb1-CTD at Ser5 and Ser7, facilitating both transcription initiation and elongation, while also activating other CDKs through T-loop phosphorylation [108]. CDK7 is frequently dysregulated in various cancers (Figure 4), including breast [109], lung [110], and haematologic malignancies, where its enhanced activity promotes uncontrolled transcription and cell proliferation [111].

Similarly, CDK10, CDK11, and CDK12 regulate transcriptional elongation, mRNA processing, and genome stability, and their dysregulation is linked to tumour proliferation, neurodegeneration, and viral replication (Figure 4 [112,113]). CDK11, in particular, acts at the intersection of transcription and mRNA processing by phosphorylating Rpb1-CTD-Ser2 and the Splicing Factor 3B1 (SF3B1), coordinating elongation and pre-mRNA splicing [114]. Its dysregulation has been linked not only to neuronal pathology but also to tumour proliferation [115] and HIV-1 replication [116] supporting its use as a multifunctional therapeutic target [112]. Similarly, CDK12 is key for transcription-replication balance and genomic stability and accordingly its loss defines a subtype of metastatic castration-resistant prostate cancer, characterized by replication stress, increased T cell infiltration, and sensitivity to immune checkpoint blockage. The parallel vulnerability of CDK12-deficient tumours to inhibition of its paralog CDK13 highlights a synthetic lethal relationship with strong therapeutic implications [113].

Collectively, transcription-associated CDKs illustrate how perturbation of RNAPII-centred phosphorylation networks can reprogramme gene-expression landscapes, thereby contributing to oncogenesis, neurodegeneration, and viral replication through mechanisms that extend beyond isolated CTD dysregulation.

## CTD phosphatases as modulators of phosphorylation network dysregulation

Although CTD kinases have historically received considerable attention, phosphatases represent an equally fundamental component of RNAPII phosphorylation control. While these enzymes directly modulate CTD phosphorylation states, their disease associations frequently reflect broader roles in signalling, chromatin regulation, and cell fate determination. Recent genomic and clinical studies have highlighted the contribution of CTD phosphatases to developmental disorders, cancer, immune dysfunction, and metabolic disease. SSU72 was initially characterized as a CTD phosphatase essential for transcriptional regulation [38], and recent studies have revealed broader functions in disease-relevant pathways in mammals (Figure 4 [39,117]). During HIV-1 infection, the viral Tat protein interacts with SSU72 and stimulates its CTD phosphatase activity, promoting the Ser5P-to-Ser2P transition at the integrated HIV-1 promoter and enhancing viral transcription [118]. In metabolic regulation, dysregulated SSU72–HNF4α signalling – HNF4α being a nuclear receptor transcription factor essential for hepatocyte identity – drives progression from steatohepatitis to hepatocellular carcinoma through aberrant HNF4α phosphorylation and hepatocyte dedifferentiation [53]. These findings establish SSU72 as a multifunctional phosphatase whose dysregulation impairs transcription factor activity and viral transcriptional control, contributing to oncogenic progression.

RPAP2, the mammalian orthologue of yeast Rtr1, has emerged as a disease-associated phosphatase with functions beyond its canonical role in transcription. In hepatocellular carcinoma (HCC), elevated RPAP2 expression correlates with reduced patient survival [11]. Normally, FBXW7 promotes RPAP2 degradation through ubiquitination after priming phosphorylation by p38 and GSK3, thereby controlling RPAP2 levels. Loss of FBXW7 leads to RPAP2 accumulation, which drives HCC cell proliferation and impairs hepatocyte differentiation [11]. In mice, hepatocyte-specific deletion of Fbxw7 causes severe hepatic cystogenesis; however, simultaneous deletion of Rpap2 completely prevents this phenotype, demonstrating that RPAP2 accumulation is responsible for the pathology [11]. These findings suggest that RPAP2 dysregulation compromises transcription initiation control and cell fate programmes, contributing to tumour development (Figure 4).

SCP1 (CTDSP1), the prototypical member of the small CTD phosphatase family, has gained attention as a disease-associated phosphatase. SCP1 is highly expressed in non-neuronal tissues of the nervous system, where it regulates REST (repressor element-1 silencing transcription factor), a master repressor of neuronal gene programmes [49,119,120]. SCP1 dephosphorylates REST at Ser861 and Ser864, preventing ubiquitination by the SCF-βTrCP ubiquitin ligase complex and subsequent proteasomal degradation, thereby stabilizing REST and maintaining its repressive activity in non-neuronal cells [121]. Dysregulation of the SCP1–REST pathway has been implicated in disease: elevated REST levels driven by SCP1 activity promote tumour growth in glioblastoma (Figure 4), whilst persistent REST upregulation following peripheral nerve injury inhibits axonal regeneration and contributes to chronic pain [122,123].

The PP2A/INTAC complex couples RNAPII dephosphorylation with transcription termination by acting on CTD-Ser2, Ser5, and Ser7 [124]. PP2A dysfunction is broadly implicated in cancer and neurodegenerative disorders (Figure 4); whether these associations arise specifically through CTD dysregulation or through broader phosphatase network disruption remains under active investigation.

Collectively, CTD phosphatases illustrate how disruption of dephosphorylation dynamics can reconfigure RNAPII-centred regulatory networks. Although these enzymes directly target the CTD, their pathological consequences frequently arise from coordinated effects on transcription factors, signalling pathways, and chromatin regulators, underscoring that phosphatase dysfunction contributes to disease through integrated phosphorylation network imbalance rather than isolated CTD defects.

## Therapeutic targeting of the RNAPII phosphorylation network

The central role of RNAPII phosphorylation dynamics in gene regulation has driven the development of diverse therapeutic strategies targeting CTD kinases, phosphatases, and CTD-interacting proteins. Rather than targeting downstream signalling pathways, these approaches seek to directly reprogramme aberrant transcriptional circuits driven by dysregulated RNAPII phosphorylation.

## Targeting transcription-associated CDKs

tCDKs have emerged as especially tractable therapeutic targets because of their defined structural pockets and their central position in controlling oncogenic transcriptional programmes. Early CDK inhibitors, such as flavopiridol, demonstrated the feasibility of transcriptional blockade but suffered from poor selectivity and dose-limiting toxicity linked to their broad CDK inhibition profiles [125,126]. Subsequent generations of small molecules have achieved greater specificity for CDK7 and CDK9, enabling more precise modulation of RNAPII phosphorylation.

CDK9 inhibitors such as enitociclib (VIP152), voruciclib, and AZD4573 induce apoptosis in transcriptionally addicted cancers by suppressing Ser2 phosphorylation and destabilizing short-lived transcripts associated with transcriptional addiction, including MYC-driven gene-expression programmes [127]. Combination regimens pairing CDK9 inhibition with venetoclax or azacitidine have shown synergy in acute myeloid leukaemia (AML) models and early-phase clinical studies [96,105]. Dinaciclib, a multikinase inhibitor with potent activity against CDK1, CDK2, CDK5, and CDK9, continues to be evaluated in small-cell lung cancer, where inhibition of RNAPII phosphorylation correlates with reduced expression of survival genes [128]. Similarly, CDKI-73 suppresses Rpb1-CTD-Ser2 phosphorylation in prostate and colorectal cancer cells, lowering *BCL2* expression and inducing transcriptional stress [129,130]. Interest in transcription-associated kinase targeting has also expanded beyond CDK7 and CDK9. In particular, CDK12 and CDK13 have emerged as promising therapeutic targets owing to their central roles in transcriptional elongation, RNA processing, and genome stability. Recent work demonstrated that CDK12/CDK13 inhibition disrupts transcriptional elongation and replication fork progression in glioblastoma models, further highlighting the therapeutic potential of targeting these kinases in transcription-dependent cancers [131]. Recent reviews have likewise emphasized the expanding clinical relevance of CDK12- and CDK13-directed therapeutic strategies across multiple tumour types [132].

At the level of transcription initiation, selective CDK7 inhibitors such as THZ1 have produced strong preclinical responses in triple-negative breast cancer and small-cell lung carcinoma through depletion of global Ser5 phosphorylation and collapse of oncogenic transcriptional circuits [133]. More recently, oral inhibitors including samuraciclib (CT7001) have advanced to clinical testing in hormone receptor – positive breast cancer, where pharmacodynamic studies confirm on-target suppression of RNAPII phosphorylation with manageable toxicity [134].

In parallel, proteolysis-targeting chimaeras (PROTACs) designed to degrade CDK9, such as dCDK9-202, exhibit potent tumouricidal activity in preclinical models, heralding a new generation of transcriptional kinase degrader strategies [135,136]. Recent studies using the compound KI-CDK9d-32 show that drugs following this strategy cause a powerful disruption in the MYC-regulated network, rapidly reducing levels of both proteins and mRNA transcripts [95]. Although most clinical progress has occurred in oncology, the centrality of tCDKs in transcriptional homeostasis suggests potential for therapeutic modulation in neurodegenerative and inflammatory disorders as well.

With regard to antiviral development, selective CDK9 PROTAC degraders have emerged as a promising tool in the field too, such as the heterobifunctional compound 9 g, which recruits Von Hippel-Lindau (VHL) protein, effectively eliminates CDK9, reduces HIV-1 RNA synthesis, and exhibits lower cytotoxicity than conventional inhibitors [137]. Among strategies targeting latency, CDK9 inhibition can transiently suppress proviral transcription, although rebound occurs upon drug withdrawal, underscoring the need to evaluate other transcription-associated CDKs for ‘block and block’ approaches [138].

## Restoring or modulating CTD phosphatase function

Whilst kinase inhibition has dominated translational development, therapeutic strategies aimed at CTD phosphatases represent an emerging frontier. In contrast to kinase blockade, which suppresses hyperactive transcriptional programmes, phosphatase-directed interventions seek to restore balanced RNAPII recycling and termination dynamics.

In CCFDN syndrome caused by biallelic CTDP1 mutations, restoration of FCP1 function represents a rational precision-medicine objective. Although no clinical trials are currently underway, experimental strategies including adeno-associated viral (AAV) gene delivery, CRISPR-mediated splice correction, and small molecules designed to stabilize residual FCP1 activity illustrate potential avenues for enzyme-restorative therapy [83]. Such precision strategies exemplify how structure-guided design and improved understanding of CTD phosphatase regulation may eventually yield enzyme-restorative treatments for rare transcriptional syndromes.

SCP1 has attracted substantial interest as a pharmacological target. Multiple inhibitor classes have been identified, including small molecules discovered by virtual screening, phosphorylation-mimic peptides isolated via phage display, and engineered antibody-mimetic scaffolds [139,140]. Notably, targeted covalent inhibitors containing α,β-unsaturated sulphone warheads selectively inactivate SCP1 through covalent modification of Cys181 at the active-site entrance, promoting REST degradation and reactivating REST-silenced genes [123]. These compounds provide compelling proof-of-concept that SCP1 inhibition can therapeutically modulate REST-dependent transcriptional networks. These studies highlight the feasibility of targeting phosphatase-mediated transcriptional repression in disease contexts driven by aberrant gene silencing.

## Targeting CTD-interacting proteins

Gene-directed strategies targeting CTD-binding proteins are transforming the therapeutic landscape of CTD-related neurodegenerative diseases. The antisense oligonucleotide ulefnersen (ION363; jacifusen) specifically reduces *FUS* mRNA and protein levels and is currently under evaluation in the multinational FUSION clinical trial for FUS-associated amyotrophic lateral sclerosis (FUS-ALS). Interim analyses demonstrate dose-dependent suppression of FUS expression in cerebrospinal fluid and favourable safety profiles [141]. Complementary open-label studies involving a small patient cohort have shown promising biomarker responses, with cerebrospinal neurofilament light chain – a marker of axonal injury – declining by more than 80% after six months of treatment [142]. These data represent a milestone in directly targeting CTD-interacting regulatory proteins implicated in phosphorylation-dependent transcriptional dysfunction.

Additional experimental strategies aimed at restoring nuclear localization of mutant FUS or limiting pathological aggregation through PARP or chaperone modulation are under investigation, reflecting the expanding scope of interventions targeting CTD-associated regulatory networks [92,143].

A precision-medicine framework is now emerging across CTD-regulatory pathologies. In neurodegeneration, routine genetic screening for *FUS* mutations is increasingly integrated into diagnostic and trial-enrolment protocols, ensuring early identification of individuals eligible for gene-specific therapies [141]. In cancer, molecular subtyping of medulloblastoma to include *CTDNEP1* mutational status has refined risk stratification and may guide the rational design of combination regimes that jointly target MYC amplification and mitotic checkpoint vulnerabilities [144]. As understanding of CTD signalling deepens, future precision interventions are likely to extend beyond individual kinases or binding proteins to encompass the broader transcriptional circuitry controlled by the RNAPII CTD.

Despite these advances, significant challenges remain, including defining tissue-specific vulnerabilities to RNAPII phosphorylation imbalance, characterizing transcriptome-wide consequences of phosphatase dysregulation, and developing selective pharmacological tools capable of safely modulating CTD phosphorylation *in vivo*. Nevertheless, the convergence of structural biology, genetics, and translational research signals a rapidly advancing field in which direct manipulation of the RNAPII phosphorylation network is becoming a tangible therapeutic strategy.

## Future directions in RNAPII phosphorylation research

Whilst the Rpb1-CTD has been extensively studied as a central regulatory platform coordinating transcription with RNA processing, far less attention has been paid to the regulatory potential of other RNAPII subunits. This CTD-centred focus contrasts with accumulating phosphoproteomic evidence indicating that most RNAPII subunits are phosphorylated *in vivo* [35]. These observations suggest that RNAPII regulation may extend beyond the canonical CTD phospho-code to encompass additional phosphorylation-dependent mechanisms embedded within the core enzyme itself. Given that these subunits are essential for viability and participate in critical structural and regulatory roles, they may harbour additional regulatory layers that remain largely unexplored.

Rpb3, the third-largest RNAPII subunit, is essential for polymerase assembly and facilitates recruitment of RNAPII to specific transcription factors. Although traditionally considered a structural component, emerging evidence indicates that Rpb3 participates in transcriptional regulatory complexes whose stability and activity may be modulated by post-translational modification. In cancer, the E3 ubiquitin ligase SPOP regulates the stability of Rpb3 and Rpb7. Loss of SPOP elevates RNAPII subunit abundance and, in the presence of active transcription factors such as Gli2, enhances assembly of transcription factor – RNAPII complexes that drive oncogenic transcriptional output [145]. Whether phosphorylation or other post-translational modifications of Rpb3 directly influence its regulatory interactions remains unknown.

Similarly, Rpb2 (encoded by POLR2B in humans) forms the active site of RNAPII together with Rpb1, and emerging evidence suggests its dysregulation contributes to cancer pathogenesis. In glioblastoma, POLR2B is frequently amplified and overexpressed, often co-amplifying with PDGFRA due to their close genomic localization on chromosome 4q12 [146]. These findings raise the possibility that quantitative or post-translational regulation of core RNAPII subunits may contribute to transcriptional reprogramming in disease. However, whether phosphorylation of Rpb2 modulates catalytic efficiency, elongation dynamics, or transcriptional fidelity remains to be determined.

Rpb6, shared by all three eukaryotic RNAPs and essential for growth in *S. cerevisiae*, is highly conserved from yeast to mammals [147]. Rpb6 was identified as one of the most prominently phosphorylated RNAP subunits and is phosphorylated by casein kinase II (CKII) at Ser2 within its N-terminal acidic region *in vitro* [148]. Beyond phosphorylation, Rpb6 possesses a short flexible N-terminal tail that interacts with the PH domain of the p62 subunit of TFIIH, and this interaction is critical for recruiting TFIIH to transcription sites, thereby coupling transcription with nucleotide excision repair across all three RNAP systems [147]. Point mutations in the Rpb6 N-terminal tail cause significant reductions in transcription of RNAPI-, RNAPII-, and RNAPIII-transcribed genes, demonstrating that this shared subunit plays multiple roles in transcription, DNA repair, and cell proliferation [147]. Whether Rpb6 phosphorylation modulates this interaction or coordinates transcription – repair crosstalk in response to stress remains an open question.

Discovering novel phosphorylation sites and regulatory modifications within non-CTD RNAPII subunits may help explain pathologies currently attributed solely to structural or assembly defects. For instance, our laboratory is currently focused on how Rpb4 phosphorylation could modulate gene Rpb4/7 function, and therefore expression [149]. Understanding how post-translational modifications regulate subunit stability, catalytic efficiency, and protein – protein interactions could reveal new therapeutic targets for transcription-related disorders. Future studies integrating structural biology, phosphoproteomics, and functional genomics will be required to define how these modifications contribute to dynamic RNAPII regulation in physiological and pathological contexts. Expanding beyond the CTD-centric framework to incorporate the regulatory roles of other subunits is essential for achieving a complete understanding of transcriptional control in health and disease.

## Data Availability

Data sharing is not applicable to this article as no data were created or analysed in this study.
